# Rear 4-min Schirmer test, a modified indicator of Schirmer test in diagnosing dry eye

**DOI:** 10.1038/s41598-022-09791-9

**Published:** 2022-04-15

**Authors:** Xin Wang, Xiaojing Fan, Yaying Wu, Yujie Mou, Jinjin Min, Xiuming Jin

**Affiliations:** 1 Eye Center of the Second Affiliated Hospital, School of Medicine, Zhejiang University, 88# Jiefang Road, Hangzhou, 310009 Zhejiang Province People’s Republic of China; 2 The Fifth People’s Hospital of Yuhang District, 60 Baojian Road, Hangzhou, 311100 People’s Republic of China

**Keywords:** Diseases, Health care, Medical research, Signs and symptoms

## Abstract

This study aims to investigate the reliability and efficacy of rear 4-min Schirmer test, as a supplement indicator, in assessing tear secretion and diagnosing dry eye. 180 participants were enrolled in this study. Schirmer test I without anaesthesia was performed once on both eyes to determine the value of normal Schirmer test. The values of tear secretion were recorded at each minute. Other examinations included the following: the ocular surface disease index (OSDI), the standard patient evaluation of eye dryness (SPEED), fluorescein stain, tear film break-up time (BUT), and Meibomian gland (MG) secretion grading. The participants were divided into dry eye (DE) group and non-dry eye (ND) group. The values of the 2-min Schirmer test, rear 3-min Schirmer test, rear 4-min Schirmer test, and 5-min Schirmer test were 5.36 ± 4.63, 5.57 ± 2.11, 7.21 ± 4.13, and 10.93 ± 6.30, respectively, in the DE group. These indicators were 8.25 ± 6.80, 2.73 ± 2.31, 7.36 ± 3.42, and 11.84 ± 6.16, respectively, in the ND group. The rear 4-min Schirmer test had a significant correlation with OSDI and SPEED in the DE group (r =  − 0.242/ − 0.183) and in the ND group (r =  − 0.316/ − 0.373). Meanwhile, the rear 4-min Schirmer test had a stronger connection with fBUT (r = 0.159) and MG secretion (r =  − 0.162) in the DE group and also had higher accuracy in diagnosing severe DE and borderline DE. In conclusion, the rear 4-min Schirmer test may be a supplement indicator in assessing tear secretion and diagnosing DE.

## Introduction

Dry eye (DE) is a common disorder on the ocular surface, and its prevalence is increasing rapidly. The incidence of DE varies from 5 to 35%^1,2^ due to geographical and ethnic differences. Approximately 30 million people worldwide have DE^3^. The International Dry Eye Workshop in 2017 defined DE as “a multifactorial disease of the ocular surface characterised by a loss of homeostasis of the tear film, and accompanied by ocular symptoms, in which tear film instability and hyperosmolarity, ocular surface inflammation and damage, and neurosensory abnormalities play etiological roles”^4^.

A stable tear film is key to a healthy ocular surface because it can protect the lubricated environment for the eyes^5^. When the tear film becomes abnormal, patients often have six main symptoms: dryness, gritty or sandy sensation, burning, redness, crust on lashes, and eyelids stuck shut in the morning^6^. The assessment of people’s symptoms is a major component in the diagnosis of DE. However, the signs of the disease do not always correlate with symptoms, thus necessitating objective ocular examinations. The most common diagnostic tests include the Schirmer test (ST)^7,8^, tear break-up time (BUT)^4^, corneal fluorescein staining (CFS)^9^, tear meniscus height^10^.

ST was first used as a diagnostic test for DE syndrome in 1903, and it can help to evaluate tear volume^11^. It is performed by placing a special filter paper at the middle and lateral thirds of the lower fornix with or without anaesthetic^8^. After 5 min, a person’s tear secretion can be measured using wetting paper. ST is always performed just once in clinical practice. However, ST has several serious disadvantages, such as discomfort, irritation, low reproducibility, requiring a long time, and inconvenience^12,13^. Because of these limitations, many efforts were made to improve the ST’s efficiency and comfort, just like to be performed this test after using anaesthesia or changing the filter paper^14^. However, these changes may not make significant improvements. With anaesthesia, a reflex component was still alive, and it may mislead physicians to judge the situation of the examinee.

The measure of tear secretion is always affected by paper irration, especially in first minute of ST^15^. Because of an adaptation of nervous system^16^, the speed of tear secretion slowed over time. This sign attracted our attention and we had a idea about how to increase the applicability and convenience of the ST. If we can remove the value of the first minute with a large difference, and then perform a weighted calculation based on the results obtained in the rear 4-min to obtain a theoretical five-minute test value, the value may be used as a supplementary data to make up for the partial shortcomings of classical ST. Our purpose was to investigate the reliability and efficacy of rear 4-min Schirmer test to help ophthalmologists diagnose DE.

## Materials and methods

### Subjects

This prospective study was performed at the outpatient clinic of the Second Affiliated Hospital of Zhejiang University School of Medicine in September 2019. The way we recruited volunteers was based on sampling of convenience. Participants attending the outpatient department of a teaching hospital attached to a medical college were enrolled in the study. The multiple rate comparison method performed with PASS version 15 was used to estimate sample size. The final sample size also included 10% dropout rate.

All participants were divided into DE and non-dry eye (ND) groups. The criteria of DE diagnosis were OSDI > 13 points and fBUT < 10 s; patients not meeting these criteria was classified into the ND group. Both eyes of per participant were included in this study. The exclusion criteria were as follows: age < 18 years; current pregnancy; eye allergies, conjunctival inflammation, corneal ulcer, eyelid inflammation, palsy, valgus, and other ocular lesions; history of wearing contact lenses within 30 days; history intraocular operation within 6 months; severe blepharitis; or severe systemic disease. This study followed the tenets of the Declaration of Helsinki and was approved by the Ethics Committee of the Second Affiliated Hospital of Zhejiang University School of Medicine (NO. 2019-307). All participants provided written informed consent after an explanation of the nature and possible consequences of the study.

### DE symptom questionnaires

#### Ocular surface disease index

The ocular surface disease index (OSDI) was a validated dry eye questionnaire that can measure the severity of DE, symptoms, functional problems, and environmental triggers queried for the past week^17^. The OSDI have been regarded as an established standard questionnaire^4^. Each OSDI answer in 12 questions was graded on a scale of 0–4. A total OSDI scores ranged from 0 to 100. The results were interpreted as follows: normal (scores 0–12) and dry eye (scores 13–100)^18^.This study used Chinese version and validated in previous studies^19,20^.

#### Standard patient evaluation of eye dryness questionnaire

The standard patient evaluation of eye dryness (SPEED) questionnaire was administered to grade the level of DE symptomology^21^. And the SPEED questionnaire is also useful in assessing dry eye symptoms in a nonclinical sample^22^. The assessment standard of the SPEED questionnaire is derived by summing the scores from the frequency and severity parts of the questionnaire for 3 months. The values of frequency and severity in the SPEED questionnaire were obtained by summing the scores of the eight items (with each rated from 0 to 4), and the total SPEED scores ranged from 0 to 28. The results were interpreted as follows: normal (scores 0) and dry eye (scores 1–28). We uesd a Chinese language version and validated in previous study^23^.

### DE symptom examinations

#### ST I

ST was performed once without topical anaesthesia, and both eyes were evaluated at the same time. The filter paper (Showa Yakuhin Kako, Tokyo, Japan) was folded and placed between the lower eyelid and the globe at the junction between the middle and lateral thirds of the eyelid. The participants were asked to close their eyes, as ST with close eyes may be more reliable in DE^24^. The test lasted 5 min, and the length of the wetted paper was directly read off the scale on the paper. Wetting was measured at 1, 2, 3, 4, and 5 min. The participants were excluded from the study if any of the scores exceeded 30 mm (the whole paper was wetted) because of the inability to give an exact measurement of the amount of wetting. The results were interpreted as follows: ≤ 5 mm: severe DE, ≤ 10 mm: borderline DE, and > 10 mm: normal tear secretion^25^. We observed multiple indicators of ST, as follows:

1. ST1: The value of the normal (5-min) ST.
2. 2-min ST: the value of the first 2 min of ST.
3. Rear 3-min ST: the value of normal ST minus that of first 2 min of ST.
4. Rear 4-min ST: the value of normal ST minus that of first min of ST.

#### Tear film BUT

Tear film instability was assessed using BUT measurements, which were obtained using the following method. TBUT tests include fluorescein tear film break-up time (fBUT) and noninvasive tear film break-up time (NIBUT). fBUT: Fluorescein was added to the tear film for both eyes, and the patients were asked to blink several times to ensure uniform distribution. The time from the last blink of the eye to the first dry spot on the tear film was measured. Three consecutive measurements were made and recorded. The average values of the three measurements were recorded. NIBUT: NIBUT was measured using Keratograph 5 M (Oculus Optikgerate GmbH, Wetzlar, Germany). The participants placed the lower jaw on the jaw rest and keep both eyes open. After successful focusing, they were asked to blink twice and look at the red point in the centre, keeping the eye open as much as possible until the next blink. Then, the device automatically detected the tear film distribution map and displayed the measured values. BUT was categorised as moderate (5–10 s) or severe (< 5 s)^26,27^.

#### Meibomian gland secretion quality grading

The quality of the Meibomian gland (MG) secretion grading was briefly assessed using a Meibomian Gland Evaluator (TearScience, Morrisville, NC, USA). A total of 15 Meibomian glands (in the nasal, middle, and temporal parts of the lower tarsus) were selected. The quality of meibum was graded as follows: 0, clear fluid; 1, thick and cloudy dropout; 2, inspissated and congealed dropout with the consistency of toothpaste; and 3, no dropout. The total scores ranged from 0 to 45.

#### CFS score

For CFS, the participants’ cornea was divided equally into five quadrants, and the score for each quadrant was recorded after fluorescein staining, as follows: 0, no punctate staining; 1, less than 30 stained points; 2, more than 30 stained points but no fusion; and 3, entirely stained with fusion. A total score of five parts was given as 0–15.

#### Sequence of DE tests

Participants completed the tests in the following order: first, each participant signed informed consent forms, completed the OSDI and SPEED questionnaires, and provided general information. Second, a Keratograph 5 M measurement was performed to obtain the NIBUT. Third, fBUT, CFS, and Schirmer I tests were performed. Finally, MG secretion was measured using MG evaluators. A ≥ 10-min intertest interval was set during which the patients were asked to rest with their eyes closed.

#### Statistics

All statistical analyses were performed using SPSS (version 24, IBM, Armonk, NY, USA) for Mac. A Kolmogorov–Smirnov test and P-P plot were used to assess the normality of the continuous variables. An independent samples t test was used to compare the parameters of the ST indicators and the demographic characteristics between the two groups. The chi-square test was used to compare the sex distribution between the two groups. Pearson’s rank-order correlation was used to identify the correlation between ST, BUT, OSDI, SPEED, MG secretion grading, and CFS. One-way ANOVA test was uesd to compare the values of four indicators of ST.

In order to investigate the reliability and efficacy of four indicators of ST, true positive rate and true negative rate were used and the chi-square test was used to compare the efficacy of four indicators of ST. The true positive rate is equal to true positive/(true positive + false negative) × 100%. The definition of true positive rate is the proportion of patients who are actually dry eye in the total number of patients who are just judged to be dry eye according to the criteria of test. The definition of patients who are actually dry eye is that their tear secretion is less than the cut-off.The true negative rate is equal to true negative/(true negative + false positive) × 100%. The true negative rate is the proportion of patients who are actually normal in the total number of persons who are just judged to be normal according to the criteria of test. The definition of normal person is that their tear secretion is more than the cut-off. The figures were created using SPSS and GraphPad Prism, version 8 (San Diego, CA). The datas are presented as mean ± standard deviation. p < 0.05 was considered statistically significant.

## Results

### Participants’ demographics and baseline characteristics

We included 360 eyes of 180 participants; of them, 240 eyes of 120 participants had DE and 120 eyes of 60 participants did not. Table 1 presents the participants’ baseline characteristics (age, sex, subjective questionnaires, and objective examinations). The objective examination results of NIBUT, fBUT, MG secretion grading, and CFS were 4.70 ± 2.28, 3.69 ± 2.22, 22.51 ± 11.68, and 0.88 ± 0.25, respectively, in the DE group and 8.43 ± 3.31, 4.22 ± 2.20, 16.32 ± 9.60, and 0.35 ± 0.06,respectively, in the ND group. The OSDI and SPEED scores were 30.12 ± 12.91 and 12.59 ± 4.63, respectively in the DE group and 6.62 ± 3.35 and 5.27 ± 2.87, respectively, in the ND group.Age was not significantly different between the two groups.Other DE-related indicators had significant differences between the two groups (p < 0.05). The normal and abnormal test results were in the Supplementary Table 1.

**Table 1:** Baseline characteristics of the subjects (Mean ± SD).

Characteristics	DE	ND	p value
Age	39.41 ± 14.05	37.62 ± 13.17	0.670
Sex (male/female)	45/75	30/30	0.034
OSDI total	30.12 ± 12.91	6.62 ± 3.35	0.029
SPEED	12.59 ± 4.63	5.27 ± 2.87	0.014
NIBUT-average (s)	4.70 ± 2.28	8.43 ± 3.31	0.038
fBUT-average (s)	3.69 ± 2.22	4.22 ± 2.20	0.040
MG secretion grading	22.51 ± 11.68	16.32 ± 9.60	0.043
CFS	0.88 ± 0.25	0.35 ± 0.06	0.020

### ST and DE group

#### Comparison among the different values of ST

**Figure 1:**
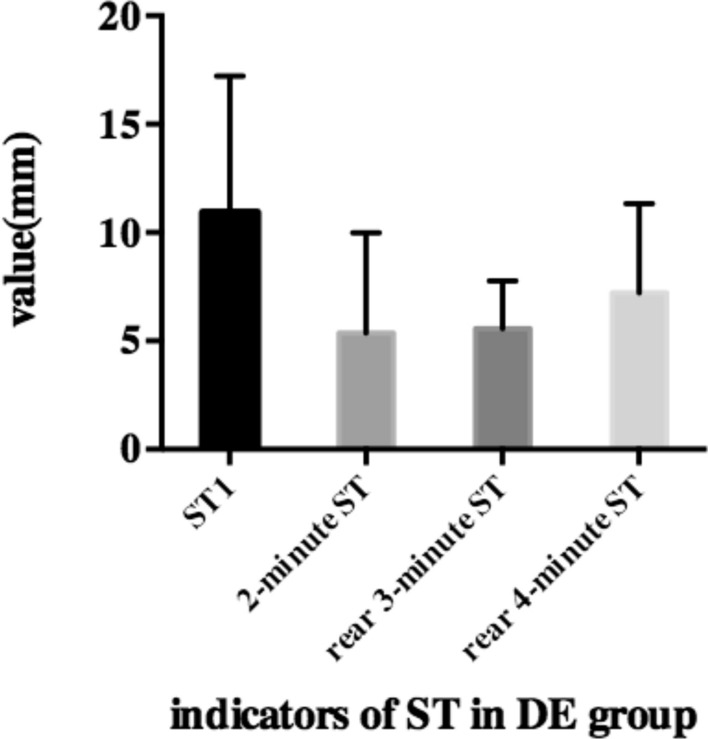
Histogram of ST1, 2-min ST, rear 3-min ST and rear 4-min ST in DE group.

**Figure 2:**
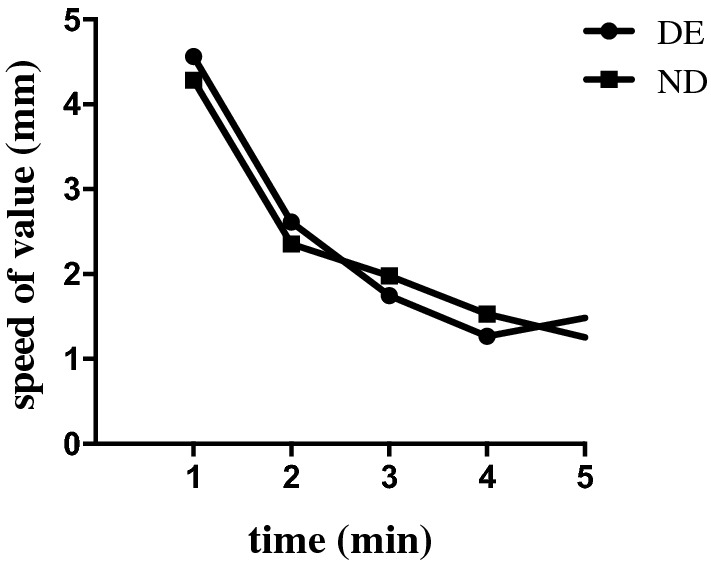
Line chart of speed of ST in each minute in DE group and ND group.

The four ST indicators were significantly different from each other in both groups. Figure 1 summarises four indicators of ST in the DE group. The values of ST1, 2-min ST, rear 3-min ST, and rear 4-min ST were 10.93 ± 6.30, 5.36 ± 4.63, 5.57 ± 2.11, and 7.21 ± 4.13, respectively, in the DE group. The four indicators were significantly different from each other (p < 0.01). Significant positive correlations were observed between ST1, 2-min ST, rear 3-min ST, and rear 4-min ST. The details about the relationships with the four indicators are listed in Table 2. We also recorded the ST results at each minute. However, we noticed a sign of tear secretion when the patients were performed ST. The speed of mean wetting per minute had been slowed down in ST. The speed was approximately 1.25–4.56 mm/min in the test, including in the DE and ND groups (Fig. 2). We observed that the speed of wetting paper in the first minute was unstable and casual in different participants. Therefore, we focused on the other indicators of ST, which removed the data from the first minute. We observed that the value of the rear of 4-min was approximately 3/5 of ST1.

**Table 2:** The relationship between four indicators in DE group.

Relationship	Value (mean ± SD)	ST1	2-min ST	Rear 3-min ST	Rear 4-min ST
Value (mean ± SD)		10.93 ± 6.30	5.36 ± 4.63	5.57 ± 2.11	7.21 ± 4.13
**ST1**	10.93 ± 6.30				
r		1.00	0.796	0.676	0.845
p		1.00	< 0.001	< 0.001	< 0.001
**2-min ST**	5.36 ± 4.63				
r		0.796	1.00	0.256	0.508
p		< 0.001	1.00	< 0.001	< 0.001
**Rear 3-min ST**	5.57 ± 2.11				
r		0.676	0.256	1.00	0.884
p		< 0.001	< 0.001	1.00	< 0.001
**Rear 4-min ST**	7.21 ± 4.13				
r		0.845	0.508	0.884	1.00
p		< 0.001	< 0.001	< 0.001	1.00

#### Different values of ST and DE symptom questionnaires

We analysed the relationship between four indicators and DE symptom questionnaires. We observed that these indicators all had a correlation with the OSDI or SPEED. Table 3 presents the details. We observed that both ST1 and rear 4-min ST had relationships with the other indicators. As we expected, ST had a weak correlation with the questionnaire scores. However, the rear 4-min ST had a higher correlation with OSDI and SPEED than ST1.

**Table 3:** The relationship between indicators of ST and OSDI, SPEED, fBUT, MG dropout grading, and CFS in DE group.

Relationship (r/p)	Value (mean ± SD)	ST1	2-min ST	Rear 3-min ST	Rear 4-min ST
Value (mean ± SD)		10.93 ± 6.30	5.36 ± 4.63	5.57 ± 2.11	7.21 ± 4.13
**OSDI**	30.12 ± 12.91				
r		− 0.198	− 0.137	− 0.081	− 0.242
p		< 0.001***	0.014*	0.155	< 0.001***
**SPEED**	12.59 ± 4.63				
r		− 0.124	− 0.059	− 0.170	− 0.183
p		0.037*	0.317	0.005**	0.002**
**fBUT**	3.69 ± 2.22				
r		0.108	0.040	0.103	0.159
p		0.024*	0.403	0.036*	0.001**
**MG secretion grading**	22.51 ± 11.68				
r		− 0.153	− 0.098	− 0.151	− 0.162
p		< 0.001***	0.022*	0.001**	< 0.001***
**CFS**	0.88 ± 0.25				
r		0.091	0.064	0.176	0.108
p		0.156	0.326	0.006**	0.090

*p < 0.05, **p < 0.01, ***p < 0.001.

#### Different values of ST and other DE examinations

The BUT was the most convenient diagnostic test used in clinical practice. The participants performed three consecutive fBUT tests and one-time NIBUT. In addition to BUT, MG secretion grading and CFS were performed. All four tests had statistical significance between the DE group and the ND group. However, we investigated the correlation between different values of ST and the other four tests. The results revealed the relationship between different ST and fBUT, MG secretion grading, and CFS in Table 3. In Table 3, we can see that the rear 4-min ST had a higher correlation with other examinations than the other three sorts of ST.

### ST and non-DE group

**Figure 3:**
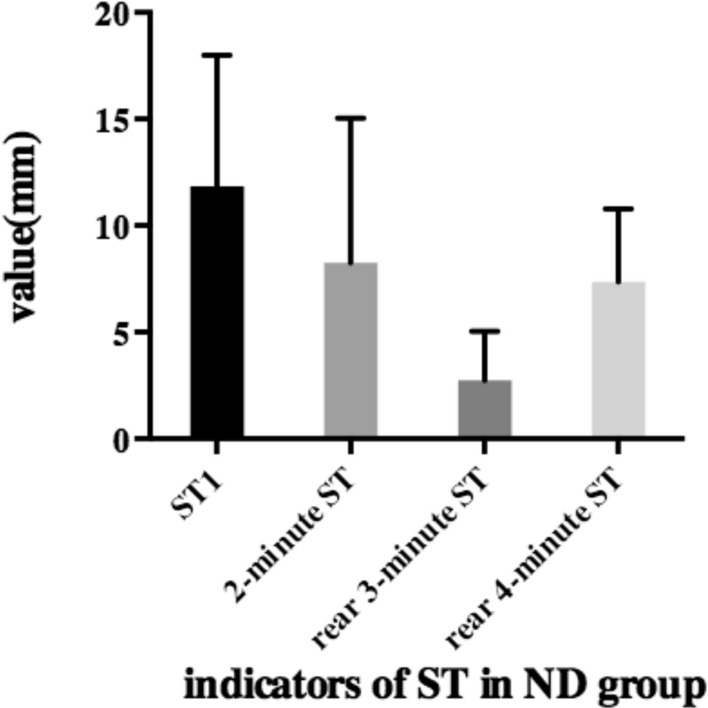
Histogram of ST1, 2-min ST, rear 3-min ST and rear 4-min ST in ND group.

The values of ST1, 2-min ST, rear 3-min ST, and rear 4-min ST were 11.84 ± 6.16, 8.25 ± 6.80, 2.73 ± 2.31, and 7.36 ± 3.42, respectively, in the ND group (Fig. 3). The four indicators were significantly different from each other (p < 0.01) in the ND group. The OSDI and SPEED scores were 6.62 ± 3.35 and 5.27 ± 2.87. Other DE tests, such as NIBUT, fBUT, MG secretion grading, and CFS, were 8.43 ± 3.31, 4.22 ± 2.20, 16.32 ± 9.60, and 0.35 ± 0.06, respectively. Table 4 shows the correlation between ST and other examinations in the ND group. We noticed the same sign in the ND group as in the DE group. The rear 4-min ST had the highest correlation with all examinations compared to the other three types of ST.

**Table 4:** The correlation between ST and other examinations in ND group.

Relationship (r/p)	Value (mean ± SD)	ST1	2-min ST	Rear 3-min ST	Rear 4-min ST
Value (mean ± SD)		11.84 ± 6.16	8.25 ± 6.80	2.73 ± 2.31	7.36 ± 3.42
**OSDI**	6.62 ± 3.35				
r		− 0.284	− 0.086	− 0.082	− 0.316
p		0.005**	0.370	0.400	0.002**
**SPEED**	5.27 ± 2.87				
r		− 0.237	− 0.174	− 0.170	− 0.373
p		0.025*	0.075	0.090	< 0.001***
**fBUT**	4.22 ± 2.20				
r		0.195	0.108	0.139	0.191
p		0.028*	0.187	0.095	0.029*
**MG secretion grading**	16.32 ± 9.60				
r		− 0.279	− 0.194	− 0.048	− 0.284
p		0.001**	0.010*	0.546	< 0.001***

*p < 0.05, **p < 0.01, ***p < 0.001.

### Rear 4-min ST may be a suitable supplement for DE diagnosis

To evaluate the four indicators in diagnosing DE, the true positive rate (TPR) was compared. The TPR of ST1, 2-min ST, rear 3-min ST, and rear 4-min ST was 101 eyes (42.08%), 75 eyes (31.25%), 68 eyes (28.33%), and 145 eyes (60.41%). We also calculated the true negative rate (TNR) in the ND group. We observed that the TNR of ST1, 2-min ST, rear 3-min ST, and rear 4-min ST was 50 eyes (41.67%), 47 eyes (39.17%), 48 eyes (40.00%), and 54 eyes (45.00%). The TPR of the four indicators showed a significant difference (p < 0.001). Based on the above results, we chose one indicator, rear 4-min ST, to be a reliable supplement for ST. We observed that the number of patients in theory whose tear secretions were less than 10 mm in 5 min accounted for 74.62% of people whose value of rear 4-min ST was lower than 6 mm. To provide the reference for the clinical diagnosis, we divided the patients into severe DE (ST ≤ 5 mm) and borderline DE (ST 5–10 mm). The value of tests in severe dry eye and borderline dry eye was in the supplementary Table 2. We compared rear 4-min ST with ST1 in subjective questionnaires and objective examinations. As shown in Table 5, the relationship between rear 4-min ST and other examinations in patients with severe DE was higher than that in patients with borderline DE. This means that in patients with severe DE, DE may be more easily diagnosed through the rear 4-min ST than classical ST.

**Table 5:** The relationship between rear 4-min ST and ST1 with examinatios.

Relationship	Severe dry eye				Borderline dry eye			
	Rear 4-min ST		ST1		Rear 4-min ST		ST1	
	r	p	r	p	r	p	r	p
**OSDI**	− 0.278	0.001**	− 0.207	0.014*	− 0.276	0.026*	− 0.274	0.038*
**SPEED**	− 0.289	< 0.001***	− 0.150	0.043*	− 0.257	0.039*	− 0.220	0.048*
**fBUT**	− 0.179	0.002*	− 0.168	0.038*	− 0.266	0.021*	− 0.207	0.05
**MGS grading**	0.196	0.015*	0.145	0.047*	0.242	0.042*	0.223	0.049*

## Discussion

No consensus exists on the diagnostic criteria for DE in different areas, even though the International Dry Eye Workshop proposed the criteria for DE diagnosis. Most ocular examinations are not a perfect means to diagnose DE^28^, and suffer from low sensitivity and specificity in detecting symptoms. One of the major reasons may be the heterogeneity of DE. However, there is no single existing test to reliably diagnose DE, and two or three tests are often required, making the process time and labor intensive. Therefore, it is crucial to simplify the process and improve the accuracy of the tests.

ST is one of the most commonly used tests for assessing tear production ability due to its low cost and simplicity. However, the value of diagnosing DE has not yet been unified with the improvements of ST over decades. Jones’^12^ prospective of basal ST was that the cut-point should be set at 10 mm/5 min; however, Van Bijsterveld^29^ thought the length of wetting paper less than 5.5 mm should be defined as deficient aqueous production. A cutoff of 5.0 mm/5 min is recommended as meaningful^4,30^. Even though ST without topical anaesthesia may cause patients discomfort, we think that reflex tear secretion is an indispensable indicator.

ST is easily influenced by many factors, such as reflex tear, paper irritation, temperature, and humidity^31,32^. Therefore, doctors have strived to improve the accuracy of ST in clinical practice. Schulze suggested altering the ST to strip meniscometry because of its rapid performance of the procedure (5 s per eye)^33^. As we know, ST without topical anaesthesia may induce reflex tearing, and reflex tear secretion is unstable. To decrease the disadvantage of irritation of filter paper, a new strip named K-Schirmer was developed in Korea, and its reliability of repeated measurements was higher than ST^34^. In Vasileios Karampatakis' research, he compared the 2- and 5-min test and evaluated the reliability and the tear secretion of the Schirmer test I in 2 min^15^. In his study, he noticed that the speed of wetting paper slowed over time. And we saw a similar sign about the speed of wetting. During the initial 1 min, the speed of tear production was faster than in the next min; this phenomenon suggested an adaptation of the central nervous system^16^. Another reason was that a few remaining tears may exist in the low fornix. For that matter, the doctor should make sure that there are no tears before the test. However, we realised that the technique and process of dipping tears were not convenient for doctors and patients. In our study, we took the ST once as usual and recorded the values of each minute to determine the elements that may be more reliable for assessing tear secretion. Our study included both patients and normal people. Therefore, we had enough samples to verify whether our selected indicator really worked.

In the long term, ST is implemented only once in clinical practice. Only one test may impair the accuracy of the results and the validity of the test. To increase the accuracy of examinations, we should take care of more than one indicator. Telles suggested measuring several dynamic wetting lengths when we gauge human tear production^35^. Therefore, we selected four indicators: ST1, 2-min ST, rear 3-min ST, and rear 4-min ST. In our study, the ST values were consistent with the OSDI and SPEED. However, the relevance was low. This was consistent with a previous report^36^. At the same time, we valued ST with other examinations, such as BUT, MG secretion grading, and CFS. BUT is considered to be the most common statistical pattern to value the stability of tear film^37^. MG secretion grading is used to assess the extent of gland obstruction^38^. The fluorescein staining score is a test that values the environment of the cornea. Similar to the results above, ST was correlated to the fBUT and MG secretion grading in the DE group. Besides ST1, we noticed an interesting phenomenon: rear 4-min ST were correlated to the fBUT and MG secretion grading in the DE group. However, it was also correlated with the fBUT and MG secretion grading in the ND group. Finally, rear 4-min ST had higher TPRin diagnosing DE. It suggested that participants in our study, regardless of which group, rear 4-min ST may be a reliable indicator in assessing tear secretion. The reasons may include adaptation of irritation, the stable speed of tear secretion and reduction in the eye’s pain of patients. For doctors, it just needs a few seconds to record the value of the first minute and fifth minute of ST, and it is able to give reliable data to evaluate tear secretion. However, some participants may be sensitive about the paper and have massive tear secretion. These people do not fit this measurement and need other tests.

In conclusion, our study found that rear 4-min ST seemed to be a better assessment of tear production. Our data indicate that rear 4-min ST < 6 mm and 5-min ST < 10 mm likely indicates DE. One of the limitations of this study is that we did not account for the drainage and evaporation of tears.

## Supplementary Information

Supplementary Table 1.
