# Supplementary material for: Rear 4-min Schirmer test, a modified indicator of Schirmer test in diagnosing dry eye
Source: Sci Rep. 2022 Apr 15;12:6272. doi: 10.1038/s41598-022-09791-9 (PMC9012812; doi:10.1038/s41598-022-09791-9)
Supplement: Supplementary file 1 — Supplementary Table 1. [file 41598_2022_9791_MOESM1_ESM.docx]

Supplementary Table 1. The results of normal and abnormal tests in participants.

| tests | abnormal（eyes） | normal（eyes） |
| --- | --- | --- |
| OSDI | 240 | 120 |
| SPEED | 330 | 30 |
| fBUT | 324 | 36 |
| MG | 278 | 82 |
| CFS | 50 | 310 |
| ST1 | 171 | 189 |
| 2-min ST | 149 | 211 |
| rear 3-min ST | 140 | 220 |
| rear 4-min ST | 211 | 149 |

Supplementary Table 2. The values of tests in severe dry eye and borderline dry eye (Mean ± SD)

| value (Mean±SD) | severe dry eye | borderline dry eye |
| --- | --- | --- |
| OSDI | 30.45±10.46 | 32.46±10.97 |
| SPEED | 12.27±2.45 | 13.69±3.47 |
| fBUT | 3.14±1.27 | 4.43±2.07 |
| MGS grading | 24.40±10.83 | 22.72±10.83 |
| ST1 | 2.96±1.67 | 7.81±1.43 |
| rear 4-min ST | 1.87±0.94 | 4.66±0.80 |
